# Dataset on estimate of intra-specific genetic variability of African yam bean (*Sphenostylis stenocarpa* (Hochst. ex A. Rich.) Harms.) based on rbcL gene marker

**DOI:** 10.1016/j.dib.2023.108944

**Published:** 2023-02-02

**Authors:** Jacob Olagbenro Popoola, Davelyne Ifechukude Eruemulor, Omena Bernard Ojuederie, Abiodun Sunday Oyelakin

**Affiliations:** a Pure and Applied Biology Programme, College of Agriculture, Engineering and Science (COAES), Bowen University, Iwo, Osun State, Nigeria; b Department of Biological Sciences, Covenant University, PMB 1023, Ota, Ogun State, Nigeria; c Department of Biological Sciences, Biotechnology Unit, Kings University, PMB 555, Ode-Omu, Osun State, Nigeria; d Food Security and Safety Focus Area, Faculty of Natural and Agricultural Sciences, North-West University, Private Bag X2046, Mmabatho 2735 South Africa; e Department of Pure and Applied Botany, College of Biosciences, Federal University of Agriculture, Abeokuta (FUNAAB), Ogun State, Nigeria

**Keywords:** African yam bean, Breeding, Genetic intra-specific diversity, Germplasm conservation, Ribulose-1,5-bisphosphate carboxylase/oxygenase large subunit (rbcL) gene, AYB, African yam bean, RbcL, ribulose-1,5-bisphosphate carboxylase/oxygenase large subunit gene.

## Abstract

African yam bean (*Sphenostylis stenocarpa* (Hochst. ex A. Rich.) Harms.) (Fabaceae) is a versatile crop of nutritional, nutraceutical, and pharmacological value widely grown for its edible seeds and underground tubers. Its high-quality protein, rich mineral elements, and low cholesterol make it a suitable source of food for age groups. However, the crop is still under-exploited and constrained by factors such as intra-specific incompatibility, low yields, indeterminate growth pattern and long gestation period, hard-to-cook (HTC) seeds, and the presence of antinutritional factors (ANFs). To efficiently utilize its genetic resources for improvement and utilization, it is necessary to understand the crop's sequence information and select promising accessions for molecular hybridization trials and conservation purposes. A total of 24 accessions of AYB were collected from the Genetic Resources center of the International Institute of Tropical Agriculture (IITA), Ibadan, Nigeria, and subjected to PCR amplification and Sanger sequencing. The dataset determines genetic relatedness among the twenty-four accessions of AYB. The data consist of partial rbcL gene sequences (24), estimates of intra-specific genetic diversity, the maximum likelihood of transition/transversion bias, and evolutionary relationships based on the UPMGA clustering method. The data identified 13 variables (segregating sites) as SNPs, 5 haplotypes, and codon usage of the species that can be explored further to advance the genetic utilization of AYB.

**Specifications Table**

**Table:** 

Subject	Biological Science
Specific subject area	Plant Science, Genetic diversity, Phylogeny, and Evolution, Bioinformatics
Type of data	Tables, Figure
How the data were acquired	Amplification of the ribulose-1,5-bisphosphate carboxylase/oxygenase large subunit (rbcL) gene, partial cds; chloroplast through PCR and DNA Sanger Sequencing. Data were analyzed using Geneious Prime 2022, DnaSP v6.12.03, and MEGAX.
Data format	Raw Analyzed
Description of data collection	A total of 25 accessions of AYB seeds were collected from the Genetic Resources center (GRC) of the International Institute of Tropical Agriculture (IITA), Ibadan. The seeds were planted in a replicate of 5 rows of five accession (25×2) and maintained for 2 weeks at the Genebank screenhouse. Accession TSs6 did not germinate and was excluded from the analysis. Twenty-four (24) accessions were assessed using rbcL primers and the genetic diversity relatedness parameters such as single nucleotide polymorphism, codon usage, and cluster analysis were estimated using MEGAX, and CodonW whereas the number of polymorphic (segregating sites), the number of haplotypes, haplotype (gene) diversity, the variance of haplotypes, nucleotide diversity and the average of nucleotide differences were calculated using DnaSP 6.0.
Data source location	The location and passport data are summarized in Table 1.
Data accessibility	Repository name: NCBI (PopSet) Accession number: 2278194789 Direct URL to data: https://www.ncbi.nlm.nih.gov/popset/?term=2278194789 All data in this paper are available at the Mendeley repository. Direct URL to data: https://data.mendeley.com/datasets/cjj62tswb2

**Value of the Data**

• The dataset provides insight into the genetic relationship among twenty-four accessions of African yam bean (AYB) – *Sphenostylis stenocarpa* using information from the partial rbcL gene sequences, nucleotide polymorphisms, haplotype diversity, and codon usage.
• The data identified 13 polymorphic sites (segregating sites) nucleotide pairs and 5 haplotypes that can be explored to broaden/enlarge the narrow genetic base of AYB via mutation breeding, nuclear techniques, and modern biotechnologies.
• Twelve accessions (TSs 1, TSs 13, TSs 24, TSs 38, TSs 49, TSs 67, TSs 98, TSs 101, TSs 303, TSs 311, TSs 331, and TSs 334) showed SNPs in the 13 variable sites.
• The SNPs can be used to generate specific markers and genetic linkage maps for AYB.
• Accession TSs 303 showed consistency in base substitution (transition and transversion) in all the 13 variable sites and accounted for 42% of the total variable sites.
• The sequence data will benefit the scientific community in the area of agriculture and researchers can deploy rbcL gene sequences in the genetic characterization of the species to infer evolutionary history toward the unbiased selection of promising accessions of AYB for breeding and conservation purposes.

## 1 Objectives

The dataset aimed to assess the partial rbcL gene sequences intra-specific variability and phylogenetic relationships among some accessions of African yam bean.

## 2 Data Description

**Fig. 1:**
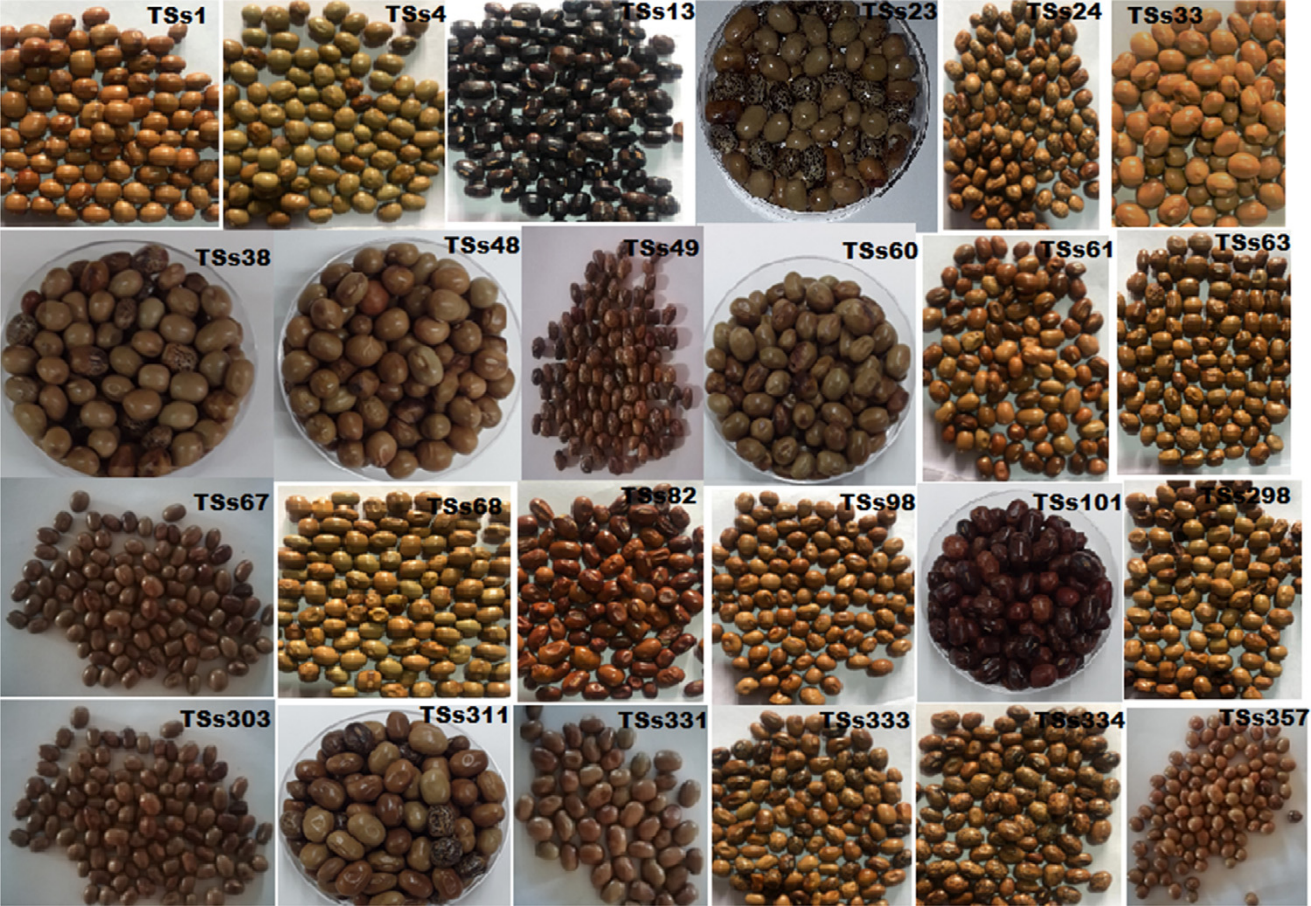
Phenotypic images of the 24 seeds of AYB used for this study.

**Fig. 2:**
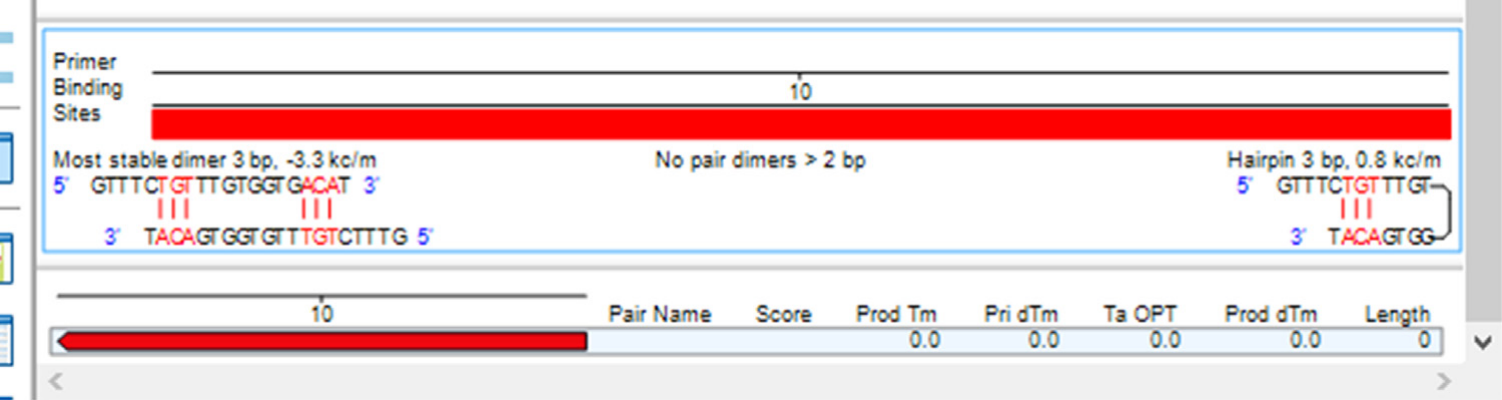
Rbcl gene structure showing primer binding sites.

**Fig. 3:**
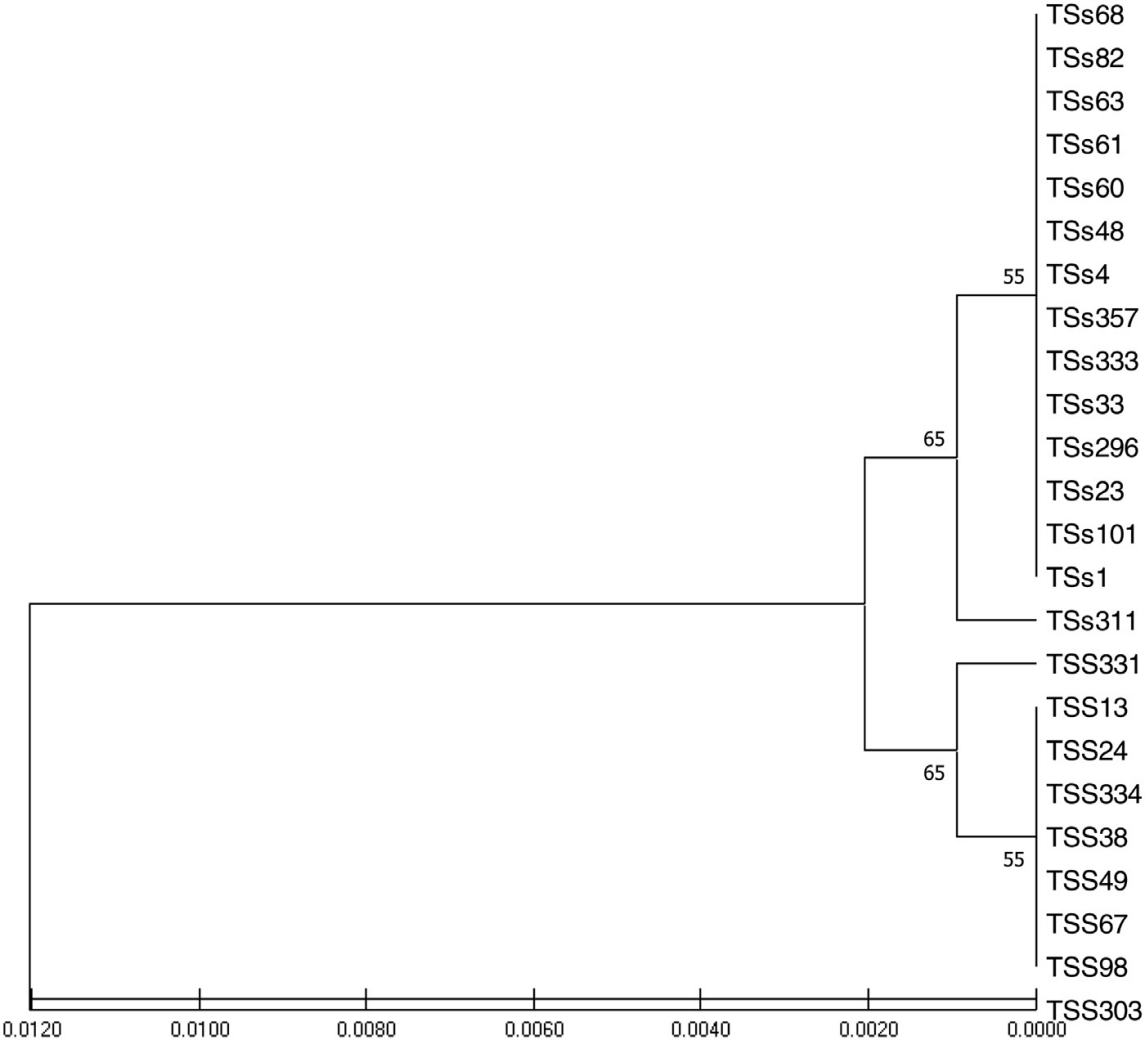
Evolutionary relationship among the twenty-four accessions of AYB using the rbcL gene marker.

African yam bean (AYB) (*Sphenostylis stenocarpa* (Hochst. ex A. Rich.) Harms.) belong to the Order: Fabale and Family Fabaceae. It is one of the nutritionally, and medicinally important orphan legumes in Central, Western, and Eastern Africa [1,2]. Its low cholesterol and high-quality protein make it a suitable source of food for obese, diabetic, and hypertensive patients [3], [4]. AYB is constrained by intra-specific incompatibility, low yields, indeterminate growth pattern and long gestation period, hard-to-cook (HTC) phenomenon, and the presence of antinutritional factors (ANFs). It is imperative to deploy marker-assisted crop improvement strategies and sequencing information to obtain more insights on AYB genetic relatedness. The data in this article presents the rbcL gene sequences of twenty-four accessions of *S. stenocarpa*. Table 1 shows the passport data and country of origin of the accessions while the phenotypic images of the accessions are presented in Fig. 1. The rbcl gene structure with the primer binding sites is shown in Fig. 2. Table 2 explains the submitted sequences to the NCBI GenBank, the assigned GenBank numbers, matched organisms, and the sequence length (bp). Single nucleotide polymorphic (SNP) of the 13 variable sites are represented in Table 3 while codon usage parameters including codon bias index (CBI), scaled chi-square, and G+C contents of coding and non-coding positions are presented in Table 4. Table 5 summarizes the codon usage and amino acid residues of the *S. stenocarpa*. The estimates of genetic diversity and Maximum Likelihood of Transition/Transversion Bias are represented in Tables 6 and 7, respectively. The evolutionary history of the taxa using the UPGMA method with five sub-clusters is presented in Fig. 3.

**Table 1:** Passport data and country of origin of the AYB accessions used for this study.

Accessions	Country of Origin	Seed shape	Seed coat color	Seed coat texture	Brilliance of seeds
TSs 1	Nigeria	Oval	Light brown	Smooth	Medium
TSs 4	Nigeria	Round	Cream	Rough	Matt
TSs 13	Nigeria	Round	Purple	Smooth	Shiny
TSs 23	Nigeria	Oblong	Light brown	Smooth	Shiny
TSs 24	Nigeria	Oblong	Cream	Wrinkled	Matt
TSs 33	Nigeria	Round	Dark brown	Wrinkled	Shiny
TSs 38	Nigeria	Oval	Dark brown	Wrinkled	Medium
TSs 48	Nigeria	Oblong	Dark brown	Wrinkled	Matt
TSs 49	Nigeria	Round	Reddish brown	Smooth	Shiny
TSs 60	Nigeria	Oblong	Reddish brown	Rough	Medium
TSs 61	Nigeria	Oval	Reddish brown	Smooth	Shiny
TSs 63	Nigeria	Round	Light brown	Rough	Matt
TSs 67	Bangladesh	Rhomboid	Dark brown	Wrinkled	Matt
TSs 68	Ghana	Round	Light brown	Smooth	Shiny
TSs 82	Nigeria	Oblong	Dark brown	Rough	Medium
TSs 98	Nigeria	Oval	Dark brown	Smooth	Shiny
TSs 101	Nigeria	Rhom	Dark brown	Wrinkled	Matt
TSs 296	Nigeria	Oval	Light brown	Smooth	Shiny
TSs 303	Nigeria	Oblong	Reddish brown	Rough	Medium
TSs 311	Nigeria	Oblong	Light brown	Smooth	Shiny
TSs 331	NA	Round	Cream	Smooth	Medium
TSs 333	NA	Round	Cream	Smooth	Medium
TSs 334	NA	Oval	Light brown	Smooth	Shiny
TSs 357	NA	Oval	Dark brown	Smooth	Medium

Source: (IITA, 2021) N/A: not available.

**Table 2:** Summary of the deposited sequences, Genbank accession numbers, matched organism, and sequence length.

Sequence Name	GenBank Accession Number	Matched Organism	% Identity	SL (bp)
*Sphenostylis stenocarpa* isolate TSs1 ribulose-1,5-bisphosphate carboxylase/oxygenase large subunit (rbcL) gene, partial cds; chloroplast	OK254173.1	*Sphenostylis stenocarpa*	100%	534
*Sphenostylis stenocarpa* isolate TSs4 ribulose-1,5-bisphosphate carboxylase/oxygenase large subunit (rbcL) gene, partial cds; chloroplast	OK254174.1	*Sphenostylis stenocarpa*	100%	534
*Sphenostylis stenocarpa* isolate TSs23 ribulose-1,5-bisphosphate carboxylase/oxygenase large subunit (rbcL) gene, partial cds; chloroplast	OK254175.1	*Sphenostylis stenocarpa*	100%	534
*Sphenostylis stenocarpa* isolate TSs33 ribulose-1,5-bisphosphate carboxylase/oxygenase large subunit (rbcL) gene, partial cds; chloroplast	OK254176.1	*Sphenostylis stenocarpa*	100%	534
*Sphenostylis stenocarpa* isolate TSs48 ribulose-1,5-bisphosphate carboxylase/oxygenase large subunit (rbcL) gene, partial cds; chloroplast	OK254177.1	*Sphenostylis stenocarpa*	100%	534
*Sphenostylis stenocarpa* isolate TSs60 ribulose-1,5-bisphosphate carboxylase/oxygenase large subunit (rbcL) gene, partial cds; chloroplast	OK254178.1	*Sphenostylis stenocarpa*	100%	534
Sphenostylis stenocarpa isolate TSs61 ribulose-1,5-bisphosphate carboxylase/oxygenase large subunit (rbcL) gene, partial cds; chloroplast	OK254179.1	*Sphenostylis stenocarpa*	100%	534
*Sphenostylis stenocarpa* isolate TSs63 ribulose-1,5-bisphosphate carboxylase/oxygenase large subunit (rbcL) gene, partial cds; chloroplast	OK254180.1	*Sphenostylis stenocarpa*	100%	534
*Sphenostylis stenocarpa* isolate TSs68 ribulose-1,5-bisphosphate carboxylase/oxygenase large subunit (rbcL) gene, partial cds; chloroplast	OK254181.1	*Sphenostylis stenocarpa*	100%	534
*Sphenostylis stenocarpa* isolate TSs82 ribulose-1,5-bisphosphate carboxylase/oxygenase large subunit (rbcL) gene, partial cds; chloroplast	OK254182.1	*Sphenostylis stenocarpa*	100%	534
*Sphenostylis stenocarpa* isolate TSs101 ribulose-1,5-bisphosphate carboxylase/oxygenase large subunit (rbcL) gene, partial cds; chloroplast	OK254183.1	*Sphenostylis stenocarpa*	100%	534
*Sphenostylis stenocarpa* isolate TSs296 ribulose-1,5-bisphosphate carboxylase/oxygenase large subunit (rbcL) gene, partial cds; chloroplast	OK254184.1	*Sphenostylis stenocarpa*	100%	534
*Sphenostylis stenocarpa* isolate TSs311 ribulose-1,5-bisphosphate carboxylase/oxygenase large subunit (rbcL) gene, partial cds; chloroplast	OK254185.1	*Sphenostylis stenocarpa*	100%	534
*Sphenostylis stenocarpa* isolate TSs333 ribulose-1,5-bisphosphate carboxylase/oxygenase large subunit (rbcL) gene, partial cds; chloroplast	OK254186.1	*Sphenostylis stenocarpa*	100%	534
*Sphenostylis stenocarpa* isolate TSs357 ribulose-1,5-bisphosphate carboxylase/oxygenase large subunit (rbcL) gene, partial cds; chloroplast	OK254187.1	*Sphenostylis stenocarpa*	100%	534
*Sphenostylis stenocarpa* isolate TSs13 ribulose-1,5-bisphosphate carboxylase/oxygenase large subunit (rbcL) gene, partial cds; chloroplast	OK254188.1	*Sphenostylis stenocarpa*	100%	534
*Sphenostylis stenocarpa* isolate TSs24 ribulose-1,5-bisphosphate carboxylase/oxygenase large subunit (rbcL) gene, partial cds; chloroplast	OK254189.1	*Sphenostylis stenocarpa*	100%	534
*Sphenostylis stenocarpa* isolate TSs38 ribulose-1,5-bisphosphate carboxylase/oxygenase large subunit (rbcL) gene, partial cds; chloroplast	OK254190.1	*Sphenostylis stenocarpa*	100%	534
*Sphenostylis stenocarpa* isolate TSs49 ribulose-1,5-bisphosphate carboxylase/oxygenase large subunit (rbcL) gene, partial cds; chloroplast	OK254191.1	*Sphenostylis stenocarpa*	100%	534
*Sphenostylis stenocarpa* isolate TSs67 ribulose-1,5-bisphosphate carboxylase/oxygenase large subunit (rbcL) gene, partial cds; chloroplast	OK254192.1	*Sphenostylis stenocarpa*	100%	534
*Sphenostylis stenocarpa* isolate TSs98 ribulose-1,5-bisphosphate carboxylase/oxygenase large subunit (rbcL) gene, partial cds; chloroplast	OK254193.1	*Sphenostylis stenocarpa*	100%	534
*Sphenostylis stenocarpa* isolate TSs303 ribulose-1,5-bisphosphate carboxylase/oxygenase large subunit (rbcL) gene, partial cds; chloroplast	OK254194.1	*Sphenostylis stenocarpa*	100%	534
*Sphenostylis stenocarpa* isolate TSs331 ribulose-1,5-bisphosphate carboxylase/oxygenase large subunit (rbcL) gene, partial cds; chloroplast	OK254195.1	*Sphenostylis stenocarpa*	100%	534
*Sphenostylis stenocarpa* isolate TSs334 ribulose-1,5-bisphosphate carboxylase/oxygenase large subunit (rbcL) gene, partial cds; chloroplast	OK254196.1	*Sphenostylis stenocarpa*	100%	534

**Table 3:** Single Nucleotide Polymorphic (SNP) position sites among the studied AYB accessions.

S/N	Positions	1	2	3	4	5	6	7	8	9	10	11	12	13
1.	TSs1	g	t	t	g	g	g	t	c	t	t	g	C	t
2.	TSs4	.	.	.	.	.	.	.	.	.	.	.	.	.
3.	TSs13	.	.	.	.	.	.	.	.	.	.	.	T	g
4.	TSs23	.	.	.	.	.	.	.	.	.	.	.	.	.
5.	TSs24	.	.	.	.	.	.	.	.	.	.	.	T	g
6.	TSs33	.	.	.	.	.	.	.	.	.	.	.	.	.
7.	TSs38	.	.	.	.	.	.	.	.	.	.	.	T	g
8.	TSs48	.	.	.	.	.	.	.	.	.	.	.	.	.
9.	TSs49	.	.	.	.	.	.	.	.	.	.	.	T	g
10.	TSs60	.	.	.	.	.	.	.	.	.	.	.	.	.
11.	TSs61	.	.	.	.	.	.	.	.	.	.	.	.	.
12.	TSs63	.	.	.	.	.	.	.	.	.	.	.	.	.
13.	TSs67	.	.	.	.	.	.	.	.	.	.	.	T	g
14.	TSs68	.	.	.	.	.	.	.	.	.	.	.	.	.
15.	TSs82	.	.	.	.	.	.	.	.	.	.	.	.	.
16.	TSs98	.	.	.	.	.	.	.	.	.	.	.	T	g
17.	TSs101	.	.	.	.	.	.	.	.	.	.	.	C	.
18.	TSs296	.	.	.	.	.	.	.	.	.	.	.	.	.
19.	TSs303	c	c	c	t	t	a	a	g	c	g	t	T	g
20.	TSs311	.	.	.	t	.	.	.	.	.	.	.	.	.
21.	TSs331	.	.	.	t	.	.	.	.	.	.	.	t	g
22.	TSs333	.	.	.	.	.	.	.	.	.	.	.	.	.
23.	TSs334	.	.	.	.	.	.	.	.	.	.	.	t	g
24.	TSs357	.	.	.	.	.	.	.	.	.	.	.	.	.

1–13: Variable sites as SNPs.

**Table 4:** Codon Usage and Codon Bias Index Analysis among the 24 AYB accessions.

S/N	Accession	ENC	CBI	SChi2	G+Cn	G+C2	G+C3s	G+Cc	G+C
1.	TSs1	n/a	0.9	0.625	n/a	0.5	0.25	0.5	0.538
2.	TSs4	n/a	0.9	0.625	n/a	0.5	0.25	0.5	0.538
3.	TSs13	n/a	0.9	0.625	n/a	0.25	0.5	0.5	0.538
4.	TSs23	n/a	0.9	0.625	n/a	0.5	0.25	0.5	0.538
5.	TSs24	n/a	0.9	0.625	n/a	0.25	0.5	0.5	0.538
6.	TSs33	n/a	0.9	0.625	n/a	0.5	0.25	0.5	0.538
7.	TSs38	n/a	0.9	0.625	n/a	0.25	0.5	0.5	0.538
8.	TSs48	n/a	0.9	0.625	n/a	0.5	0.25	0.5	0.538
9.	TSs49	n/a	0.9	0.625	n/a	0.25	0.5	0.5	0.538
10.	TSs60	n/a	0.9	0.625	n/a	0.5	0.25	0.5	0.538
11.	TSs61	n/a	0.9	0.625	n/a	0.5	0.25	0.5	0.538
12.	TSs63	n/a	0.9	0.625	n/a	0.5	0.25	0.5	0.538
13.	TSs67	n/a	0.9	0.625	n/a	0.25	0.5	0.5	0.538
14.	TSs68	n/a	0.9	0.625	n/a	0.5	0.25	0.5	0.538
15.	TSs82	n/a	0.9	0.625	n/a	0.5	0.25	0.5	0.538
16.	TSs98	n/a	0.9	0.625	n/a	0.25	0.5	0.5	0.538
17.	TSs101	n/a	0.9	0.625	n/a	0.5	0.25	0.5	0.538
18.	TSs296	n/a	0.9	0.625	n/a	0.5	0.25	0.5	0.538
19.	TSs303	n/a	1	2	n/a	0.667	0.667	0.667	0.538
20.	TSs311	n/a	1	1.5	n/a	0.5	0	0.417	0.462
21.	TSs331	n/a	1	1.5	n/a	0.25	0.25	0.417	0.462
22.	TSs333	n/a	0.9	0.625	n/a	0.5	0.25	0.5	0.538
23.	TSs334	n/a	0.9	0.625	n/a	0.25	0.5	0.5	0.538
24.	TSs357	n/a	0.9	0.625	n/a	0.5	0.25	0.5	0.538

ENC = Effective number of codons, CBI = Codon bias index, Schi2 = Scaled Chi Square, G+Cn = G+C content at noncoding positions, G+C2 = G+C content at second coding position, G+C3s = G+C content at (synonymous) third coding positions, G+Cc = G+C content at coding region, G+C=G+C content in the genome (whole region), n/*a* = not available.

**Table 5:** Amino acid residues/Codon Usage of *S. stenocarpa*.

Codon	Freq	RSCU	Codon	Freq	RSCU	Codon	Freq	RSCU	Codon	Freq	RSCU
Phe-UUU	4	1.33	Ser-UCU	4	3.43	Tyr-UAC	11	1.69	Cys-UGU	2	1.33
UUC	2	0.67	UCC	1	0.86	UAC	2	0.31	UGC	1	0.67
Leu-UUA	3	1.13	UCA	0	0	TER UAA	0	0	TER UGA	0	0
UUG	5	1.88	UCG	1	0.86	UAG	0	0	Trp-UGG	2	1
CUU	4	1.5	Pro-CCU	7	2.33	His-CAU	1	1	Arg-CGU	3	1.8
CUC	0	0	CCC	2	0.67	CAC	1	1	CGC	1	0.6
CUA	2	0.75	CCA	2	0.67	Gln-CAA	4	2	CGA	3	1.8
CUG	2	0.75	CCG	1	0.33	CAG	0	0	CGG	1	0.6
Ile-AUU	4	1.5	Thr-ACU	11	2.75	Asn-AAU	3	1.2	Ser-AGU	1	0.86
AUC	4	1.5	ACC	3	0.75	AAC	2	0.8	AGC	0	0
AUA	0	0	ACA	2	0.5	Lys-AAA	7	1.4	Arg-AGA	2	1.2
Met-AUG	1	1	ACG	0	0	AAG	3	0.6	AGG	0	0
Val-GUU	5	1.82	Ala-GCU	6	1.71	Asp-GAU	7	1.56	Gly-GGU	8	1.88
GUC	1	0.36	GCC	2	0.57	GAC	2	0.44	GGC	1	0.24
GUA	4	1.45	GCA	4	1.14	Glu-GAA	9	1.5	GGA	4	0.94
GUG	1	0.36	GCG	2	0.57	GAG	3	0.5	GGG	4	0.94

Freq = Observed frequency, RSCU = Relatively Synonymous Codon Usage.

**Table 6:** Genetic diversity among twenty-four accessions of *Sphenostylis stenocarpa*.

Index	Value
No. of nucleotides	24
No. of polymorphic (segregating sites)	13
No. of haplotypes	5
Haplotype (gene) diversity	0.594
Variance of Haplotype diversity + SD	0.0070 ± 0.083
Nucleotide diversity (Pi)	0.00382
Average number of nucleotide differences (k)	2.040

SD = Standard deviation.

**Table 7:** Maximum Likelihood Estimate of Transition/Transversion Bias.

From\To	A	T	C	G
A	–	8.0374	8.0374	8.9253
T	8.0374	–	8.9253	8.0374
C	8.0374	8.9253	–	8.0374
G	8.9253	8.0374	8.0374	–

The estimated Transition/Transversion bias (R) is 0.56. The nucleotide frequencies are *A* = 25.00%, T/*U* = 25.00%, C = 25.00%, and *G* = 25.00%.

## 3 Experimental Design, Materials and Methods

### 3.1 Acquisition of AYB Seeds

A total of 25 accessions of AYB seeds were collected from the Genetic Resources center (GRC) of the International Institute of Tropical Agriculture (IITA), Ibadan, Nigeria, while twenty-four accessions were used for this study (Table 1). One of the accessions (TSs6) did not germinate and was excluded from the analysis.

### 3.2 Genomic DNA Extraction

Genomic DNA was extracted from the sample leaves (Table 1) using Zymo Research Quick-DNA Plant/Seed Miniprep Kit (Catalogue No. D6020) following the manufacturer's instructions.

### 3.3 PCR Amplification, Visualization, and Purification

PCR reactions for the rbcL regions were amplified using the composition as follows: 12.5 µL of NEB OneTaq 2X Master Mix with Standard Buffer (Catalogue No. M0482S), 2 µL of genomic DNA (10–30 ng/µl), 0.5 µL RbcL 1F (5′-ATGTCACCACAAACAGAAAC-3′) and 724R (5-GTAAAATCAAGTCCACCGCG-3) (10 µM), and 9.5 µL of nuclease-free water (Catalogue No. E476) to a final volume of 25 µL. The PCR reaction conditions and the integrity of the PCR samples followed standard procedures. The rbcl gene structure and primer binding sites are illustrated in Fig. 2.

### 3.4 Post-PCR Purification and Sequencing Analysis

PCR products were cleaned using an enzymatic method (ExoSAP) whereas the PCR amplicons were sequenced at Inqaba biotechnical Industries (Pty) Ltd, South Africa using the Nimagen, Brilliant Dye™ Terminator Cycle Sequencing Kit V3.1, BRD3-100/1000 according to manufacturer's instructions.

### 3.5 Data Analysis

Sequences were cleaned and aligned using default settings in Geneious Prime 2022 [5]. Aligned sequences were then imported into MEGA to estimate transition/transversion bias (R). Analyses were conducted using the Maximum Composite Likelihood model including +2nd+3rd+Noncoding codon positions. The phylogenetic relationship among the accessions was inferred using the UPGMA method [6]. Genetic diversity indices such as numbers of polymorphic/segregating sites (S), haplotype number (h), haplotype diversity (Hd), nucleotide diversity (π), average number of nucleotide differences (k), Single Nucleotide Polymorphic (SNP) position sites and codon bias index were estimated using DnaSP 6.0 [7]. Amino acid residues and codon usage indices were estimated using CodonW as implemented on a public Galaxy server (https://galaxy.pasteur.fr/).

## Ethics Statement

The seeds were acquired under the Standard Material Transfer Agreement (SMTA) of the International Treaty on Plant Genetic Resources. No: SMTA-00AF05-00BO89-201210 collected from the Genetic Resources center (GRC) of the International Institute of Tropical Agriculture (IITA), Ibadan, Nigeria. The seeds were cultivated and leaves were harvested under standard conditions specified by the agreement. The experiment in this article does not involve animals.

## CRediT authorship contribution statement

**Jacob Olagbenro Popoola:** Conceptualization, Methodology, Supervision, Validation, Writing – original draft. **Davelyne Ifechukude Eruemulor:** Supervision, Formal analysis. **Omena Bernard Ojuederie:** Writing – review & editing. **Abiodun Sunday Oyelakin:** Writing – review & editing.

## Declaration of Competing Interest

The authors declare that they have no known competing financial interests or personal relationships that could have appeared to influence the work reported in this paper.

## Data Availability

Partial rbcL gene sequences of 24 AYB (Original data) (NCBI (Popset)). Data on RbCl genetic diversity of AYB (Original data) (Mendeley Data).
